# GFA-Net: Geometry-Focused Attention Network for Six Degrees of Freedom Object Pose Estimation

**DOI:** 10.3390/s25010168

**Published:** 2024-12-31

**Authors:** Shuai Lin, Junhui Yu, Peng Su, Weitao Xue, Yang Qin, Lina Fu, Jing Wen, Hong Huang

**Affiliations:** 1 Shandong Non-Metallic Materials Institute, Jinan 250031, China; i53linshuai@126.com; 2 Key Laboratory of Optoelectronic Technology and Systems of the Education Ministry of China, Chongqing University, Chongqing 400044, China; yujunhui@cqu.edu.cn (J.Y.); supeng@stu.cqu.edu.cn (P.S.); aarynfu@163.com (L.F.); 3 Beijing Institute of Space Machinery and Electronics, Product Testing Center, Beijing 100094, China; xuewt4672@163.com (W.X.); yangqin138250@163.com (Y.Q.)

**Keywords:** pose estimation, RGB image, deep learning, geometric feature, dense correspondences

## Abstract

Six degrees of freedom (6-DoF) object pose estimation is essential for robotic grasping and autonomous driving. While estimating pose from a single RGB image is highly desirable for real-world applications, it presents significant challenges. Many approaches incorporate supplementary information, such as depth data, to derive valuable geometric characteristics. However, the challenge of deep neural networks inadequately extracting features from object regions in RGB images remains. To overcome these limitations, we introduce the Geometry-Focused Attention Network (GFA-Net), a novel framework designed for more comprehensive feature extraction by analyzing critical geometric and textural object characteristics. GFA-Net leverages Point-wise Feature Attention (PFA) to capture subtle pose differences, guiding the network to localize object regions and identify point-wise discrepancies as pose shifts. In addition, a Geometry Feature Aggregation Module (GFAM) integrates multi-scale geometric feature maps to distill crucial geometric features. Then, the resulting dense 2D–3D correspondences are passed to a Perspective-n-Point (PnP) module for 6-DoF pose computation. Experimental results on the LINEMOD and Occlusion LINEMOD datasets indicate that our proposed method is highly competitive with state-of-the-art approaches, achieving 96.54% and 49.35% accuracy, respectively, utilizing the ADD-S metric with a 0.10d threshold.

## 1. Introduction

Object pose estimation seeks to locate objects and determine their six degrees of freedom (6-DoF) pose relative to a camera [1]. Accurate pose estimation is crucial for applications [2] including robotic grasping [3], autonomous navigation [4,5,6], and augmented reality experiences [7]. Methods employing only RGB data have gained significant interest for real-world deployments, considering the unrestricted spatial sensing capabilities and ubiquity of RGB cameras. Estimating the 6-DoF object pose from a single RGB image, however, presents challenges owing to severe occlusions and variations in cluttered backgrounds.

Traditional approaches typically correlate 2D object images with their corresponding 3D models for object pose estimation [8,9,10]. Template matching, for instance, estimates pose by comparing a given image against a library of pre-rendered model projections from various viewpoints. The Speeded Up Robust Features (SURF) [11] method extracts keypoints and generates descriptors from an input image, then employs matching algorithms to compare these feature descriptors with those of the target object. The resulting correspondences are then utilized by the Perspective-n-Point (PnP) algorithm to calculate the pose. A significant limitation of these methods, however, is their dependence on hand-crafted textures for matching, rendering them susceptible to failure with texture-less objects and vulnerable to image variations.

Advances in deep learning, particularly with convolutional neural networks (CNNs), have derived significant progress in object pose estimation. Prevalent methods either directly regress a 6-DoF object pose from the image [12,13] or establish 2D–3D correspondences [14,15], and employ a RANSAC-based PnP algorithm to obtain pose information. Direct methods offer greater speed compared to indirect methods but often necessitate pose optimization to improve accuracy; whereas, indirect methods predict predefined sparse 2D keypoints to construct 2D–3D correspondences and recover object pose utilizing the PnP algorithm. Indirect methods generally exhibit superior performance due to the relative ease of keypoint localization. Nevertheless, keypoint-based methods struggle with occluded objects, as keypoints may be obscured. To address this limitation, coordinates-based methods, which establish dense 2D–3D correspondences [16,17], have been introduced to achieve more robust and accurate results. These methods predict the 3D coordinate for each object pixel to construct these dense correspondences. The density of these correspondences, however, inevitably introduces challenges related to efficiency and increased computational cost compared to sparse methods.

A challenge is that pose estimation is influenced solely by the object’s area, significantly affecting the network’s ability to learn object pose. Therefore, significant research focuses on enhancing the network’s ability to extract deeper features. Methods employing depth images demonstrate improved performance due to the geometric information in such images. Hodan et al. [18] introduced surface region attention from 3D models as supplementary supervision. However, while these methods depend on depth or model information for added geometric features, they overlook extracting representative features from RGB images, despite how the rich geometric and texture information they contain are crucial for pose estimation.

To address these challenges, we propose a novel approach, the Geometry-Focused Attention Network (GFA-Net), designed for RGB-only scenarios. This approach establishes dense 2D–3D correspondences and employs a PnP module for pose calculation. GFA-Net extracts the key geometric and texture features through a Pixel-wise Feature Attention (PFA) module and a Geometry Feature Aggregation Module (GFAM). The PFA module captures pixel-level feature changes by integrating spatial coordinate information into attention maps. Then, the GFAM aggregates multi-scale geometric features generated by the PFA, focusing on capturing representative object features, thereby enhancing the network’s ability to understand the pose of the object. Finally, the dense correspondences and geometric attention weights produced by the GFAM are input to the PnP module for pose determination. In summary, this paper’s principal contributions are as follows:

- The proposed GFA-Net centers on extracting the key geometric features from RGB images, thereby promoting both pose learning capacity and the efficiency of pose computation.
- Pixel-wise Feature Attention (PFA) incorporates coordinate-based positional data, enabling the network to focus on the object region and develop a robust sense of spatial relationships.
- A Geometry Feature Aggregation Module (GFAM) aggregates multi-scale features and identifies relationships among prominent geometric features.
- On the LINEMOD dataset, the proposed method attains 96.54% in the ADD metric (0.1d) on the LINEMOD dataset, and achieves 49.35% on the Occlusion LINEMOD dataset.

## 2. Related Work

Single RGB image pose estimation techniques can generally be classified into two main categories: direct and indirect methods. In the following, a concise account of the baseline approach in this paper, along with the aforementioned two methods, will be provided. Moreover, the role and contribution of attention mechanisms in the extraction of geometric features will be examined and reviewed.

### 2.1. Baseline Method

Coordinates-Based Disentangled Pose Network (CDPN), designed for real-time 6-DoF object pose estimation in RGB images, operates in a structured manner. Firstly, a two-stage method extracts the target region: rapid detection obtains rough results of the target’s center and size, followed by fixed-size segmentation for pixel extraction. Then, the Dynamic Zoom In (DZI) [16] operation scales the object based on sampled data. After that, the backbone network extracts features, and the “rotation head” predicts 3D coordinates and confidence with MCC Loss. Subsequently, a threshold filters pixels, and the PnP algorithm calculates the rotation matrix after mapping coordinates. Meanwhile, the Scale-Invariant Translation Estimation (SITE) network predicts the translation vector with its specific computation and loss function. Finally, the rotation matrix (by PnP) and translation matrix (by SITE) are integrated into CDPN to achieve the 6-DoF pose estimation.

### 2.2. Direct Methods

Direct approaches seek to estimate 6-DoF pose from a single image without recourse to the PnP algorithm. PoseCNN [13] employs a CNN architecture to directly regress 6-DoF pose from an input image; however, the non-linearity of rotation space complicates rotation estimation. To address this issue, Kehl et al. [12] introduced a method that converts the regression problem into a classification problem by discretizing the pose space into classifiable viewpoint bins. This discretization, however, derives a coarse result, typically requiring pose optimization for accurate 6-DoF pose. Manawadu and Park [19] proposed a two-stage strategy that decouples the projection scale ambiguity between object translation and focal length, resulting in a significant improvement in the accuracy of 6-DoF pose estimation. Sun et al. [20] proposed a dynamic keypoint selection algorithm that identifies keypoints in the foreground feature map for the extraction of geometric and appearance information. Su et al. [21] proposed a novel CNN-based approach that uses single-channel synthetic images for 6-DoF pose regression and applies domain adaptation to bridge the gap between real and synthetic images, improving accuracy and speed. Jin et al. [22] provided the initial translation using a depth map, and then predicted the rotation and refined the translation by combining the ROI and the original image. This method leveraged depth information to significantly reduce the search space for the subsequent module.

### 2.3. PnP-Based Methods

PnP-based methods predict keypoints in an image and establish 2D–3D correspondences by pairing these keypoints with their corresponding 3D coordinates. Then, a RANSAC-based PnP algorithm calculates the pose. This approach effectively reduces the problem of 6-DoF pose estimation to one of keypoint detection. Zhang et al. [23] introduced a real-time processing system for 6-DoF pose recovery from RGB images. Rad et al. [15] employed segmentation to identify the accurate object region, then regressed the projections of the 3D bounding box from this identified region. Single regression approaches, however, often exhibit limited accuracy. To circumvent direct keypoint regression, some methods [14,24] generate heatmaps for pose determination. The pixel-wise voting network [25] leverages pixel-wise unit vectors to vote for keypoint positions, demonstrating resilience to occlusion across various applications. Dede et al. [26] incorporated a classifier block to evaluate predefined object aspects, thereby enhancing keypoint selection.

In recent work, there has been a growing preference for coordinate-based methods, which predict dense rather than sparse correspondences [27,28,29]. For instance, Brachmann et al. [30] employed a random forest to predict dense 3D–3D correspondences from RGB-D images. This coordinate-based strategy has also been extended to the RGB domain; a regularized automatic context regression framework [31] was developed to jointly predict object labels and coordinates from a single RGB image. Nigam et al. [32] utilized segmentation to identify the object region and then regressed the 3D coordinates from the detected region. More recently, Coordinates-based Disentangled Pose Network (CDPN) [16] has adopted a strategy of separately predicting rotation and translation, utilizing a coordinate-based approach for rotation and direct regression from the image for translation. While coordinate-based methods generally exhibit better performance due to the use of dense correspondences, this density can lead to efficiency issues and increased processing time in the PnP module.

### 2.4. Attention-Based Methods

Visual attention mechanisms focus on relevant aspects of input data to improve network efficiency in data processing and highlight areas that enhance the performance of results. These attention-based approaches find widespread application across various visual tasks, including image detection [33], classification [34,35], and segmentation [36]. The Squeeze-and-Excitation Network [34] determines the significance of individual feature channels, concentrating on those that significantly contribute to the output. Spatial attention methods [37] allow the network to prioritize regions containing representative information. The Channel-Spatial Attention Pose Estimation Network [38] (CSA6D) incorporates channel-spatial attention modules to enhance the fusion capabilities of RGB image and point cloud data. Although it can enhance the integration of RGB images and point cloud data, in the presence of complex scene occlusions or segmentation errors, it is still affected by incorrect pixels. Its advantage, however, is that it can utilize multi-modal feature information to improve the target pose estimation ability in various scenarios. Song et al. [39] introduced a point-wise attention module. By integrating channel attention modules and MLPs, it enhances the feature extraction capabilities of RGB-D images, enabling precise location of key features. Nevertheless, its computational complexity is relatively high, which leads to low efficiency when processing large-scale point cloud data under limited resources. The Edge-Attention Pose Estimation Net (EANet) [40] incorporates edge information into the network, thus enhancing performance on objects with little texture. However, its performance in estimating the pose of symmetrical targets is restricted and it is more applicable to industrial scenarios involving low-texture components. Stevšič et al. [41] utilized spatial attention to enhance the efficiency of pose estimation. The spatial attention mechanism performs well in pose estimation with occlusions, but it is affected by the initial pose error and requires multiple iterations. During the iterative refinement, it can effectively utilize spatial details and ignore occluded parts.

## 3. Proposed Method

### 3.1. Architecture Overview

This section introduces the Geometry-Focused Attention Network (GFA-Net), a novel framework for accurate pose estimation. Firstly, GFA-Net employs an object detector to localize the object in a single RGB image. Then, geometric feature extraction generates representative geometric features and produces dense weighted 2D–3D correspondences. Finally, a PnP module leverages these correspondences to compute the 6-DoF pose.

**Figure 1:**
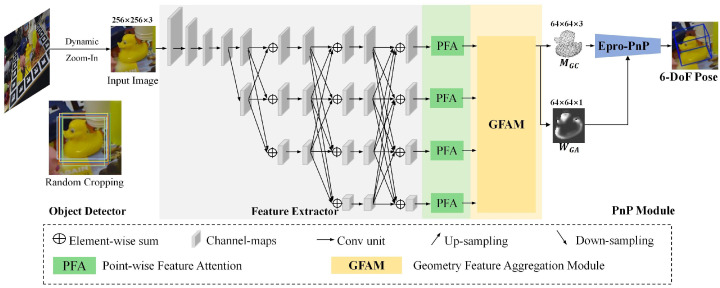
The overall architecture of GFA-Net, including object detector, feature extractor, the Point-wise Feature Attention, the Geometric Feature Aggregation Module and a PnP module.

As depicted in Figure 1, the object detector identifies the object region, receiving the original RGB image and producing a bounding box for the detected object. The original image is then cropped and augmented utilizing DZI to randomly adjust the cropped image size. This zoomed-in image is then input to the geometric feature extractor. The geometric feature extractor, responsible for deriving geometric features, comprises three components: (1) feature extractor, (2) Point-wise Feature Attention (PFA), and (3) Geometry Feature Aggregation Module (GFAM).

### 3.2. Feature Extractor

For discriminative feature extraction from RGB images, GFA-Net employs a High-Resolution Network (HRNet) [42] as its backbone. HRNet maintains high-resolution representations throughout feature extraction, producing multi-scale feature maps. These multi-scale feature maps are then passed to the Point-wise Feature Attention module for further geometric feature extraction.

### 3.3. Point-Wise Feature Attention

**Figure 2:**
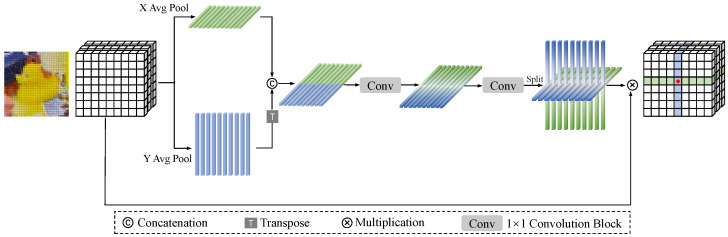
Illustration of PFA, which captures pixel-wise feature attention.

Considering that subtle changes in pose result in pixel-level image changes, Point-wise Feature Attention (PFA) is developed to establish strong spatial relationships among multi-scale feature maps, thereby capturing these changes. The architecture of PFA is illustrated in Figure 2.

The PFA can achieve accurate positioning by assigning weights to the pixels of key parts of an object and capturing the changes in their positions and values via feature map operations. It also integrates spatial and channel information to generate weights and emphasizes the changes in pixels of key structures, thereby enhancing feature representation. In complex scenarios, it adjusts pixel weights and deduces poses based on the changes in key pixels to enhance robustness. Moreover, it concentrates on key pixels and conducts fine-grained computations to capture pose-related pixel-level feature changes and utilize resources efficiently.

To avoid the position information loss associated with 2D global pooling, PFA encodes spatial features by decomposing 2D global pooling into two independent 1D feature encoding operations. For a given input feature I, two average pooling kernels (H,1) and (1,W) are utilized to encode the horizontal and vertical coordinates, respectively. Thus, the output *x* at height h can be formulated as

(1) $$\begin{array}{c} x^{h}=\frac{1}{W}\sum_{0⩽i⩽W}I\left(h,i\right) \end{array}$$

The output *x* at width *w* can be computed by

(2) $$\begin{array}{c} x^{w}=\frac{1}{H}\sum_{0⩽j⩽H}I\left(j,w\right) \end{array}$$

The synchronization of coordinate information from two separate directions is essential for generating point-wise attention. A concatenation operation merges $x^{h}$ and $x^{w}$, after which the combined features are processed through a shared 1 × 1 convolutional transformation function $F_{r}\left(\cdot \right)$. The resulting transformed features can be represented as

(3) $$\begin{array}{c} y=\delta (F_{r}\left(Concat\left[x^{h},x^{w}\right]\right)) \end{array}$$

where Concat[·,·] denotes the concatenation operation along the spatial dimension, and δ indicates a non-linear activation function. The intermediate feature map $y\in R^{\frac{C}{r}*\left(H+W\right)}$ represents spatial information from both horizontal and vertical directions, with r denoting the reduction ratio. Then, a separation operation splits the $y\in R^{\frac{C}{r}*\left(H+W\right)}$ into two separate features: $y^{h}\in R^{\frac{C}{r}*H}$ and $y^{w}\in R^{\frac{C}{r}*W}$. Two separate 1 × 1 convolutional transformation functions, $F_{h}\left(\cdot \right)$ and $F_{w}\left(\cdot \right)$, are then employed to adjust the channels of $y^{h}$ and $y^{w}$ to match the channel of the input *I*. These functions can be expressed as

(4) $$\begin{array}{cc} f^{h} & =\gamma (F_{h}\left(y^{h}\right)) \end{array}$$

(5) $$\begin{array}{cc} f^{w} & =\gamma (F_{w}\left(y^{w}\right)) \end{array}$$

where γ(·) represents the sigmoid function. The outputs $f^{h}$ and $f^{w}$ are then used as attention weights. Finally, the output of PFA can be written as the following:

(6) $$\begin{array}{c} O=I*f^{h}*f^{w} \end{array}$$

### 3.4. Geometry Feature Aggregation Module

**Figure 3:**
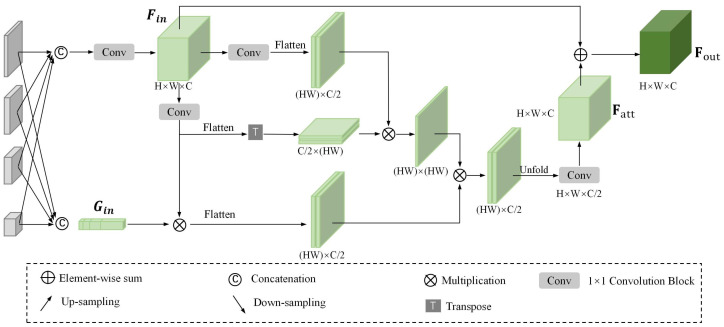
The detailed structure of Geometry Feature Aggregation Module (GFAM).

A Geometry Feature Aggregation Module is proposed to aggregate multi-scale feature maps and extract useful geometric features. GFAM extracts multi-scale spatial geometry and identifies long-range dependencies among the feature maps at different scales. Figure 3 details the GFAM architecture.

The input feature maps $I_{i}\;\left(i=1,2,3,4\right)$ vary in scale; therefore, $I_{i}\;\left(i=2,3,4\right)$ are up-sampled to match the scale of $I_{1}$ prior to concatenation, defined as follows:

(7) $$\begin{array}{c} I_{c}=Concat\left(I_{1}+f_{21}\left(I_{2}\right)+f_{31}\left(I_{3}\right)+f_{41}\left(I_{4}\right)\right) \end{array}$$

where Concat(·) denotes the concatenation in channel dimension, and $f_{ij}\left(\cdot \right)$ represents the up-sampled operation that up-samples the input of scale *i* to scale *j*. Take $f_{ij}\left(I_{i}\right)$ for instance, the input representation $I_{i}$ is up-sampled through (i−j) stride-2 3 × 3 convolutions, and $I_{c}\in R^{C\times H\times W}$ indicates the concatenated representation with abundant geometric information. A 1 × 1 convolutional function $F_{f}\left(\cdot \right)$ is used to fuse these multi-scale features that can be expressed as follows:

(8) $$\begin{array}{c} F_{in}=F_{f}\left(I_{c}\right) \end{array}$$

Following multi-scale fusion, the internal relationships in the fused features need to be extracted. Two separate 1 × 1 convolutional transformations generate the key matrix *K* and the query matrix *Q*, which are utilized for attention map construction. A transpose operation is applied during query matrix generation. Matrix generation proceeds as follows:

(9) $$\begin{array}{cc} K & =\theta (F_{in}) \end{array}$$

(10) $$\begin{array}{cc} Q & =\phi {\left(F_{in}\right)}^{T} \end{array}$$

where θ(·) denotes the 1 × 1 convolutional transformation function and flattening operation, and ϕ(·) represents the key matrix generation and flattening operation. Reshape operation is used before multiplication. These convolutional functions reduce channel number from input channel C to output channel C/2. The transformed features $Q\in R^{HW*\frac{C}{2}}$ and $K\in R^{\frac{C}{2}*HW}$ are then multiplied to get the weight matrix $w_{ij}$, which can be expressed by

(11) $$\begin{array}{c} w_{ij}=Q*K \end{array}$$

where $w_{ij}\in R^{HW*HW}$ represents pixel-level inherent correlations.

The global average pooling operation is adopted to generate channel-wise attention, which can be written as

(12) $$\begin{array}{c} g_{i}=\frac{1}{H_{i}*W_{i}}\sum_{j=1}^{H}\sum_{k=1}^{W}I_{i}\left(j,k\right) \end{array}$$

Channel-wise attention, denoted as $g_{i}\in R^{C*1*1}$, is derived from input feature maps $I_{i}\;\left(i=1,2,3,4\right)$ through concatenation and Squeeze-and-Excitation. This operation involves a hidden layer that performs dimensionality reduction and expansion utilizing two fully connected (FC) layers. $G_{in}$ is calculated as follows:

(13) $$\begin{array}{c} G_{i}=\sigma \left(W_{1}\left(W_{0}*g_{i}\right)\right) \end{array}$$

(14) $$\begin{array}{c} G_{in}=Concat\left(\sum_{i=1}^{4}G_{i}\right) \end{array}$$

where σ refers to the sigmoid function, $W_{0}\in R^{\frac{C}{r}*C}$ and $W_{1}\in R^{C*\frac{C}{r}}$. $G_{in}$ and $F_{in}$ are multiplied and a 1 × 1 convolutional function is applied to produce value matrix $V\in R^{\frac{C}{2}*C}$, which is defined as

(15) $$\begin{array}{c} V=\lambda (G_{in}*F_{in}) \end{array}$$

where λ(·) represents the value matrix generation and flattening operation. The aggregated features $F_{att}\in R^{H*W*C}$ can be expressed by

(16) $$\begin{array}{c} F_{att}=\tau \left(V*w_{ij}\right) \end{array}$$

where τ denotes a convolutional function that turns the channel C/2 to channel *C*, and the unfolding operation is utilized here. The final output of GFAM can be written as

(17) $$\begin{array}{c} F_{out}=F_{att}+F_{in} \end{array}$$

The $F_{out}$ contains geometric coordinate map ($M_{GC}$) and geometry attention weights ($W_{GA}$). These two features can be obtained by subsequent separation.

### 3.5. Solving Dense Correspondences with Weights

#### 3.5.1. Building Dense Correspondences $\left(M_{2D-3D}\right)$

Dense correspondences are required to compute pose utilizing the PnP module. Here, GFA-Net predicts $M_{GC}$ to establish these correspondences. $M_{GC}$ encodes coordinate information, mapping each 2D pixel belonging to the object to a corresponding 3D coordinate in the real world. Dense correspondence maps are then constructed by pairing each 2D pixel with its corresponding 3D coordinates in $M_{GC}$. Critically, the accuracy of MGC is essential for generating reliable dense correspondences. Therefore, $M_{GC}$ is supervised during training with an L1 loss, defined as follows:

(18) $$\begin{array}{c} Loss=L1({\tilde{M}}_{GC}-M_{GC}) \end{array}$$

where ${\tilde{M}}_{GC}$ and $M_{GC}$ represent the ground truth map and predicted map, respectively.

#### 3.5.2. Dense Correspondence Resolution Through PnP

This work employs EPro-PnP for the calculation of dense correspondences and the generation of 6D pose. This method permits the assignment of weights to correspondences, aligning with the predicted $W_{GA}$. The $W_{GA}$ represents supplementary geometric data for the dense correspondences ($M_{2D-3D}$). Increased weighting is applied to regions rich in geometric features, which further applies the efficiency of the pose estimation process.

## 4. Experiments and Discussion

### 4.1. Dataset

#### 4.1.1. LINEMOD Dataset [10]

**Figure 4:**
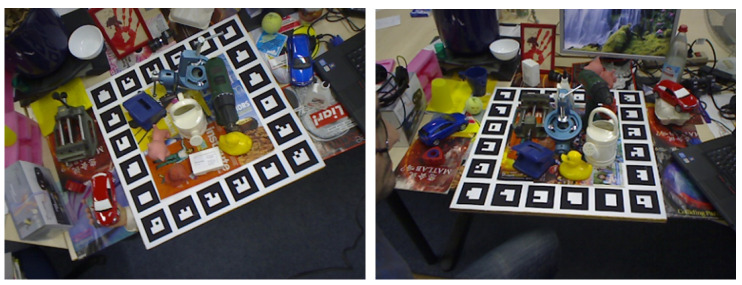
The example images of LINEMOD dataset (**left**) and the Occlusion LINEMOD dataset (**right**). Only the complete object is labeled on the LINEMOD dataset, while all the occluded object are labeled on the Occlusion dataset.

The LINEMOD dataset represents a widely adopted benchmark for 6-DoF object pose estimation. As shown on the left in Figure 4. Comprising 13 object instances with varying geometric and textural properties, the dataset comprises a range of conditions, including cluttered scenes, objects with low texture, and differences in lighting. Each object is represented by over 1000 annotated images with corresponding poses. Approximately 15% of these RGB images are allocated for training, while the remaining 85% are reserved for testing. To address potential overfitting, 1000 synthetic images are incorporated into the training set.

#### 4.1.2. Occlusion LINEMOD Dataset [31]

The Occlusion LINEMOD dataset, a subset of the LINEMOD dataset, introduces a greater degree of occlusion. As shown on the right in Figure 4. This dataset consists of 1214 images, each containing multiple annotated objects with corresponding poses, represented by significant occlusion. The model evaluated on this dataset is trained on the original LINEMOD dataset, supplemented by 10,000 rendered synthetic images per object. During training, the backgrounds of these synthetic images are randomly substituted with indoor scenes from the PASCAL VOC2012 dataset.

#### 4.1.3. YCB-Video Dataset

The YCB-Video dataset presents a basic challenge for pose estimation tasks, featuring occlusion, clutter, and several symmetrical objects. This dataset incorporates 92 videos, totaling over 110,000 real images. Of these, 80 videos are designated for training, with the remainder allocated for testing. In addition to the real training images, synthetic data generated utilizing physically based rendering (pbr) is employed during training.

### 4.2. Implementation Details

A Linux server equipped with an NVIDIA TITAN RTX featuring 24 GB of memory represented the experimental platform. The deep learning framework utilized is PyTorch 1.5.0, implemented utilizing Python 3.7. The target detector is consistent with the baseline model, which is a YOLOv3 target detection model, and in order to have a fairer comparison, this paper directly uses the YOLOv3 weight file that has been trained by the baseline model for target detection. Optimization was performed with RMSProp, utilizing a batch size of 32. Training for both the LINEMOD and Occlusion LINEMOD datasets comprised 320 epochs, starting with a learning rate of 0.0001, which was halved every 80 epochs.

### 4.3. Evaluation Metrics

#### 4.3.1. ADD(-s) Metric [10]

The ADD(-s) metric, prevalent in numerous established algorithms [16,25], represents an evaluation criterion. Object points are transformed according to both estimated and ground truth poses. The calculation of the average distance between these transformed points determines the accuracy of the estimation. An estimation is considered correct if the average distance declines below a predefined threshold. The ADD(-s) metric represents the percentage of correct estimations across the entire test dataset. For objects exhibiting symmetry, the ADD(-s) metric [15] is employed. This variant calculates the average distance based on the nearest point distance.

(19) $$\begin{array}{cc} ADD & =\frac{1}{n}\sum_{i=1}^{n}∥\left(Rx_{i}+T\right)-\left(\tilde{R}x_{i}+\tilde{T}\right)∥ \end{array}$$

(20) $$\begin{array}{cc} ADD-S & =\frac{1}{n}\sum_{i=1}^{n}min_{x_{j}\in \Delta }∥\left(Rx_{i}+T\right)-\left(\tilde{R}x_{i}+\tilde{T}\right)∥ \end{array}$$

where *n* represents the number of object’s vertices, Δ denotes the symmetric object model, and [R,T] and $\left[\tilde{R},\tilde{T}\right]$ indicate the ground truth and predicted pose, respectively.

#### 4.3.2. Two-Dimensional Projection Metric

This metric calculates the average pixel difference between two projections of a 3D model, one based on the estimated pose and the other on the ground truth pose. An estimation is considered accurate if this pixel difference is below 5 pixels. The metric represents the percentage of accurate estimations across the entire dataset.

#### 4.3.3. The 2 cm 2° Metric [43]

A pose estimation is considered accurate if the translational error is less than 2 cm and the rotational error is less than 2°. Specifically, an estimation is classified as correct if the mean distance is below a predefined threshold. The 2 cm 2° metric reflects the percentage of correct estimations in the entire test dataset.

### 4.4. Performance Comparison

#### 4.4.1. Performance on the LINEMOD Dataset

To assess its effectiveness, GFA-Net’s performance is compared against state-of-the-art (SOTA) approaches, with detailed results presented in Table 1 and Table 2. The methods utilized in the comparison can be categorized into three groups: direct, keypoint-based, and coordinate-based [16]. DenseFusion [43], DeepIM [44], and SMOC-Net [45] exemplify direct methods. Representing keypoint-based methods are PVNet [25], HybridPose [46], and a knowledge distillation method [45]. Finally, CDPN [16], GDR-Net [17], and the method proposed by Chen et al. [28,29] constitute the coordinate-based methods.

Table 1 offers a detailed comparison between our network and state-of-the-art algorithms on the LINEMOD dataset. The results demonstrate the algorithm’s performance across 13 object categories with varying sizes and textures. Indirect methods outperform direct methods as keypoint and coordinate detection offer greater accuracy than direct pose regression. Direct methods struggle with rotation due to the non-linearity of the rotation space, which reduces network generalizability. PVNet and HRPose, which predict eight keypoints in RGB images, demonstrate lower performance compared to coordinate-based methods due to sparse keypoint representation. Both GDR-Net and CDPN adopt a coordinate-based strategy and utilize an object detector for image preprocessing. This object detection promotes the performance of pose estimation for small objects such as ape and can. Dense correspondence, compared with sparse methods, brings more information for PnP calculations, thereby directly improving pose estimation performance. GDR-Net incorporates surface region attention from the 3D model, offering supplementary geometric information that compensates for the absence of such information in the depth image. Therefore, GDR-Net achieves superior performance compared to CDPN and confirms the significance of geometric information.

GFA-Net centers on the extraction of geometric features, derived through PFA and GFAM modules. PFA extracts pixel-level object motion, while GFAM extracts the key geometric features from the image. Crucially, these geometric features are derived solely from a single RGB image, without recourse to supplementary models or depth data. Moreover, the extracted geometric features generate attention weights for dense correspondences, thereby enhancing the efficiency of the PnP solution. This geometric feature extraction allows GFA-Net to surpass the performance of other methods.

To evaluate the proposed algorithm, two stringent thresholds for the ADD metric are employed: 2D Projection (5 pixels) and 2°, 2 cm. The algorithm demonstrates competitive performance even with more demanding ADD metric thresholds, such as 0.02d and 2°, 2 cm. This robust performance under stricter evaluation criteria is attributable to GFA-Net’s enhanced sensitivity to coordinate information and its ability to extract critical geometric features through PFA and GFAM.

#### 4.4.2. Performance on the Occlusion LINEMOD Dataset

Occlusion significantly degrades pose estimation performance, necessitating evaluation on the Occlusion LINEMOD dataset. Table 3 presents a comparative analysis with other SOTA RGB-based methods on this dataset. The results presented utilize performance metrics from other methods under consistent training conditions. PoseCNN, which derives pose information directly from RGB images, produces a coarse estimate and requires subsequent Iterative Closest Point (ICP) algorithm for better accuracy. PVNet leverages vector-field heatmaps to address the limitations of sparse keypoints, thereby achieving superior performance compared to PoseCNN. GDR-Net incorporates geometric features through surface region attention derived from the 3D model, while HybridPose integrates edge and symmetry information to address occlusion challenges. Therefore, both GDR-Net and HybridPose demonstrate improved performance.

GFA-Net likewise prioritizes geometric feature extraction; specifically, it centers on regions exhibiting salient object geometry, even with incomplete object visibility. In addition, in contrast to sparse-based approaches, the dense correspondences demonstrate greater resilience to occlusion, enabling the PnP module to compute the 6-DoF pose even under partial occlusion conditions. Therefore, GFA-Net achieves performance similar to other leading methods.

#### 4.4.3. Performance on the YCB-Video Dataset

Table 4 offers a detailed comparative analysis of GFA-Net and other RGB-based methods on the YCB-Video dataset. In contrast to the LINEMOD dataset, YCB-Video instances contain significant RGB texture information, rendering methods reliant on geometric features from CAD models or depth images less effective. PVNet and GDR-Net opt to train the network model independently for each object instance, and this training tactic confers certain advantages over the other three methods. In contrast, DEEPIM and CosyPose elect to incorporate pose refinement to enhance overall accuracy. Owing to its geometry-centric approach, GFA-Net progressively acquires the prominent geometric features of the objects during the training phase. The assimilation of geometric features augments the network’s concurrent learning efficiency, enabling it to adeptly handle the learning of multiple object instances.

### 4.5. Ablation Study

The ablation experiments are performed to verify the effect of each module proposed in our algorithm. Experiments are carried out on the LINEMOD dataset. GFA-Net can be divided into three parts: Baseline, PFA, and GFAM. The role of these modules in pose estimation are demonstrated by gradually overlaying them on Baseline. Experiments use three different thresholds in ADD(-S) to analyze the effects of proposed modules. The detailed results are demonstrated in Table 5.

The pixel-wise changes in images are related to pose change, so the network needs to be sensitive to such changes. The problems occur when the threshold of the ADD metric is lowered to 0.05d and 0.02d. In our work, PFA is explored to build the strong spatial awareness among multi-scale feature maps. The architecture of PFA focuses more on information at the pixel level, encoding operations in two different directions are applied to extract pixel-wise features. Therefore, PFA allows the network to locate the position of the object more accurately and capture subtle changes in rotation.

For analysis of the PFA described in Section 3.3, the feature extractor was added with a PFA module. To study the potential of PFA, this module was applied to the feature extractor’s multi-scale feature maps, enabling the capture of coordinate information therein. Integrating the PFA module with the Baseline resulted in improved ADD metrics of 0.67, 1.46, and 1.67, with more significant improvements observed in ADD(-S) metrics at lower thresholds (0.05d and 0.02d). This enhancement can be attributed to the module’s capacity for encoding coordinate information in multi-scale feature maps. The advantages of PFA are further highlighted by performance gains under stricter ADD metric thresholds.

GFAM, by aggregating and fully exploring the relationships in multi-scale feature maps, generates $W_{GA}$ incorporating key object geometry. To confirm the value of generating geometric features in pose estimation, GFAM was added to the Baseline. The resulting experiments demonstrated improved ADD metrics of 0.82, 2.37, and 2.43, respectively. GFAM extracts the geometric features embedded in the multi-scale feature maps and thoroughly explores the interrelations between these maps. Object geometry offers significant improvements to pose estimation.

PFA enhances the network’s pose change detection capabilities by incorporating coordinate information in the feature maps, while GFAM effectively aggregates multi-scale feature maps, thoroughly exploring their relationships and extracting geometric features. These two modules proposed improve the capabilities of the network from two different perspectives, resulting in significant improvements of 1.20, 3.24, and 4.64, respectively.

### 4.6. Visualization

#### 4.6.1. Visualization of $W_{GA}$

**Figure 5:**
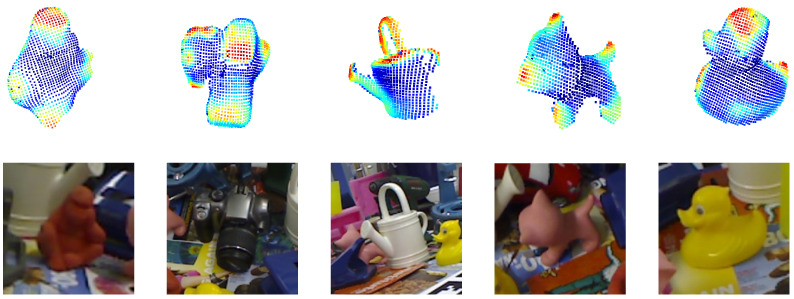
The highlighted coordinates and corresponding RGB images. The coordinates with heatmaps are the multiplication of Geometry Attention Weights ($W_{GA}$) and Geometry Coordinates Map ($M_{GC}$). Key object geometric areas are highlighted in red color.

To carry out a more intuitive representation of the geometric features extracted by GFAM, visualization experiments were conducted. $W_{GA}$ exhibits rich geometric attention, contributing to an increase in overall accuracy. As depicted in Figure 5, $W_{GA}$ accurately highlights regions containing the key geometric features of the object, represented by the red areas. These highlighted regions, rich in geometric features, emphasize the significance of geometric feature extraction.

These highlighted regions in the $W_{GA}$ mechanism signify a greater weighting applied to the respective dense correspondences. This weighting, accordingly, enhances the computational efficiency of pose determination for such dense correspondences.

#### 4.6.2. Visualization of Pose Estimation

This experiment employs the reprojection of a 3D bounding box with eight vertices. The two three-dimensional bounding boxes are reprojected onto the RGB image using, respectively, the estimated pose and the ground truth pose. The three-dimensional bounding box with green lines is the reprojection with the ground truth pose, while the box with blue lines is the estimated pose.

**Figure 6:**
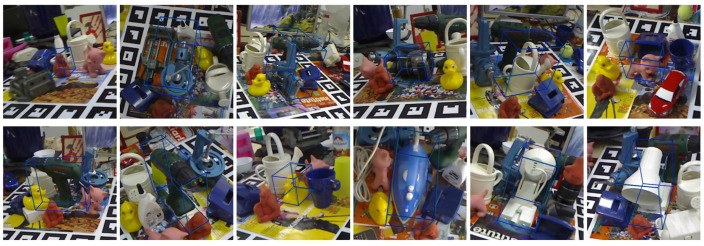
Visualizations of 6-DoF results on the LINEMOD dataset. The green boxes are the reprojection of ground truth poses, and the blue boxes represent the predicted poses.

**Figure 7:**
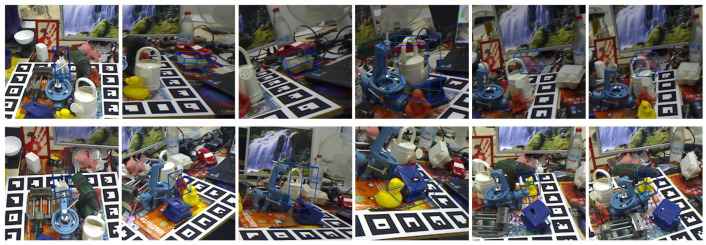
Visualizations of 6-DoF results on the Occlusion LINEMOD dataset, which contains extreme occlusion.

Figure 6 demonstrates the close alignment of the two three-dimensional bounding boxes. The initial images in both the first and second rows depict projections of object ape from two perspectives, and the network can accurately estimate its 6-DoF pose in each. Figure 7 illustrates a scenario with occlusion of the target objects; however, the GFA-Net successfully predicts poses even under these challenging conditions.

## 5. Conclusions

6-DoF object pose estimation is essential for robotic grasping, autonomous driving, and augmented reality. Considering their unrestricted spatial perception and ubiquity, RGB cameras, and thus RGB-based pose estimation methods, are of significant importance in practical applications. However, occlusions and background clutter present significant challenges for these methods. To address these challenges, we introduce a novel approach for 6-DoF object pose estimation. Point-wise Feature Attention captures fine-grained, pixel-level attention across multi-scale feature maps. Then, GFA captures and aggregates intrinsic geometric feature information from these multi-scale features. GFA-Net demonstrates competitive performance against SOTA methods on the LINEMOD and Occlusion LINEMOD datasets.

Future work will concentrate on leveraging attention mechanisms for more efficient geometric feature extraction and on developing more general and efficient geometric feature extraction modules.

## Figures and Tables

**Table 1:** Detailed comparison of the LINEMOD dataset using the 0.10d threshold of ADD(-S) as metric. Numbers highlighted in bold represent the best results.

Methods	Ape	Benc.	Cam	Can	Cat	Dril.	Duck	Egg.	Glue	Hole.	Iron	Lamp	Phone	Ave.
⊛ Densefusion [43]	79.50	84.20	76.50	86.60	88.80	77.70	76.30	99.90	99.40	79.00	92.10	92.30	88.00	86.20
⊛ DeepIM [44]	77.00	97.50	93.50	96.50	82.10	95.50	77.70	97.10	99.40	85.80	98.30	97.50	87.70	88.60
⊛ SMOC-Net [45]	85.60	96.70	97.20	**99.90**	95.00	**100.00**	76.00	98.30	99.20	45.60	99.90	98.90	94.00	91.30
⊕ PVNet [25]	43.62	**99.90**	86.86	95.47	79.34	96.43	52.58	99.15	95.66	81.92	98.88	99.33	92.41	86.27
⊕ Hybridpose [46]	63.10	**99.90**	90.40	98.50	89.40	98.50	65.00	**100.00**	98.80	89.70	**100.00**	99.50	94.90	91.30
⊕ Guo et al. [47]	76.20	96.70	92.00	94.00	88.60	94.80	74.70	99.30	97.70	82.20	93.20	96.80	89.60	90.40
⊞ CDPN [16]	64.38	97.77	91.67	95.87	83.83	96.23	66.76	99.72	**99.61**	85.82	97.85	97.89	90.75	89.86
⊞ GDR-Net [17]	76.29	97.96	95.29	98.03	93.21	97.72	80.28	99.53	98.94	91.15	98.06	99.14	92.35	93.69
⊞ Chen et al.-v1 [28]	-	-	-	-	-	-	-	-	-	-	-	-	-	95.80
⊞ Chen et al.-v2 [29]	-	-	-	-	-	-	-	-	-	-	-	-	-	96.36
★ GFA-Net (Ours)	**87.90**	98.55	**98.73**	**99.51**	**95.01**	97.62	**87.04**	99.81	99.42	**94.58**	99.08	**99.81**	**97.92**	**96.54**

⊛ Direct Methods, ⊕ Keypoint-Based Methods, ⊞ Coordinate-based Methods, ★ Proposed Method.

**Table 2:** Comparison of the LINEMOD dataset in more and stricter metrics, including ADD (0.02d and 0.05d) metric, 2D Projection (5 pixels), and 2°, 2 cm.

Method	ADD(-S)			2D Proj.	2° 2 cm
	0.02d	0.05d	0.10d		
CDPN [16]	29.10	69.50	89.86	98.10	-
GDR-Net [17]	35.60	76.00	93.69	-	67.10
Chen et al.-v1 [28]	44.81	81.96	95.80	-	80.99
Chen et al.-v2 [29]	43.77	81.73	96.36	-	-
GFA-Net	49.01	84.31	96.54	99.23	83.22

**Table 3:** Detailed comparison of the Occlusion LINEMOD dataset, using the 0.10d threshold of ADD(-S). Numbers highlighted in bold represent the best results.

Methods	Ape	Can	Cat	Driller	Duck	Eggbox	Glue	Hole.	Average
PoseCNN [13]	9.60	45.20	0.90	41.40	19.60	22.00	38.50	22.10	24.90
PVNet [25]	15.81	63.30	16.68	25.24	**65.65**	50.17	49.62	39.67	40.77
Hu et al. [48]	19.20	65.10	18.90	69.00	25.30	52.00	51.40	45.60	43.30
Hybridpose [46]	20.90	**75.30**	24.90	**70.20**	27.90	52.40	53.80	54.20	47.50
Guo et al. [47]	26.90	54.70	**32.90**	52.90	27.00	50.00	56.90	54.50	44.50
GDR-Net [17]	**41.30**	71.10	18.20	54.60	41.70	40.20	59.50	52.60	47.40
GFA-Net(Ours)	35.32	59.38	32.18	46.46	42.03	**55.47**	**66.89**	**57.03**	**49.35**

**Table 4:** Comparison on the YCB-Video dataset using the AUC of ADD(-S) metric. Refi. indicates whether the method uses pose refinement. P.E. indicates whether the method is trained with 1 pose estimator for the whole dataset or 1 per object (N objects in total).

Method	Refi.	P.E.	AUC of ADD(-S)	AUC of ADD-S
PoseCNN [13]		1	61.3	75.9
PVNet [25]		N	73.4	-
GDR-Net [17]		N	84.4	91.6
GDR-Net [17]		1	80.2	89.1
DeepIM [44]	✓	1	81.9	88.1
CosyPose [49]	✓	1	84.5	89.8
GFA-Net		1	**84.7**	**92.4**

**Table 5:** The ablation study on the LINEMOD dataset, exploring the role two separate modules play in the network.

	Module			ADD(-S)		
	Baseline	PFA	GFAM	0.10d	0.05d	0.02d
1	✓			95.34	81.07	44.37
2	✓	✓		96.01	82.53	46.04
3	✓		✓	96.16	83.44	46.80
4	✓	✓	✓	**96.54**	**84.31**	**49.01**

## Data Availability

The original contributions presented in the study are included in the article; further inquiries can be directed to the corresponding authors.
